# A global dataset of enteric methane mitigation experiments with lactating and non-lactating dairy cows conducted from 1963 to 2022

**DOI:** 10.1016/j.dib.2023.109459

**Published:** 2023-07-28

**Authors:** Mary Beth de Ondarza, Alexander N. Hristov, Juan M. Tricarico

**Affiliations:** a Paradox Nutrition, LLC, P.O. Box 105, West Chazy, NY 12992, USA; b Department of Animal Science, Pennsylvania State University, 109 AVBS Building, University Park, PA 16802, USA; c Dairy Management Inc., 10255 W. Higgins Road, Suite 900, Rosemont, IL 60018, USA

**Keywords:** Ruminant, Enteric methane, Greenhouse gas mitigation, Dairy sustainability, Feed additives, Nutrition

## Abstract

A dataset of descriptive information was compiled from 213 peer-reviewed scientific publications that focused on dairy cow experiments and measured enteric methane emissions. This dataset was primarily based on the bibliography used by Arndt et al. (2022), with the addition of studies conducted from 2019 to 2022. The articles were identified for inclusion in the dataset using the “Web of Science Core Collection” database, using various combinations of search terms related to methane, dairy, cattle, rumen, ruminant, energy balance, energy metabolism, energy partitioning, and enteric emissions. For inclusion in the dataset, studies had to be written in English and provide information on enteric methane emission, as well as report feed dry matter intake along with measures of variance. Both continuous and crossover design studies were included, resulting in a comprehensive dataset with 797 records (rows) and 162 variables (columns). The variables cover various aspects such as publication information, experimental design, animal description, methane measurement method, and diet nutrient composition. Additionally, when available, the dataset includes treatment means and measures of variance for feed dry matter intake, rumen fermentation parameters, nutrient digestibility, nitrogen excretion, milk yield, milk components, as well as enteric methane, carbon dioxide, and hydrogen emissions. Researchers can use this dataset to assess the effectiveness of different enteric methane mitigation strategies and their impact on milk yield and other essential dairy cow nutrition and performance variables. Furthermore, it offers the opportunity to explore potential interactions between nutrients and feed additives.

**Specifications Table**

**Table:** 

Subject	Animal Science
Specific subject area	Dairy cow enteric methane emissions, milk and milk components, nutrient digestibility, and rumen parameters associated with consumed nutrients and feed additives.
Type of data	Table
How the data were acquired	Data was acquired from scientific publications. The bibliography used by Arndt et al. [1] (1963 to 2018) was supplemented with a literature search to cover the period between 2019 and 2022. The literature search on the Web of Science Core Collection database used the term “methane” in combination with “dairy”, “cattle”, “rumen”, or “ruminant”. Additionally, the search term “rumen” was used in combination with “energy balance”, “energy metabolism”, or “energy partitioning”. A final search was conducted with the terms “enteric” and “dairy”. The dataset containing 797 records (rows) and 162 variables (columns) was constructed in Microsoft Excel using the 213 peer-reviewed publications identified.
Data format	Analyzed
Description of data collection	Publications were identified through literature search and met the following inclusion criteria: English language, detailed production responses, and, at a minimum, enteric methane and feed dry matter intake means with measures of variance. Information from 797 treatments was organized in rows and 162 columns containing variables, including experimental design, animal definition, methane measurement method, dietary nutrients, and treatment responses.
Data source location	Research studies conducted worldwide, including Australia, Belgium, Brazil, Canada, Chile, China, Denmark, Finland, France, Germany, India, Ireland, Italy, Japan, Mexico, New Zealand, Norway, Poland, South Africa, Sweden, Switzerland, The Netherlands, U.K., Uruguay, and U.S.A.
Data accessibility	Repository name: ScholarSphere (institutional repository managed by Penn State University Libraries) Direct URL to data: https://doi.org/10.26207/hcjx-8w62

## Value of the Data

• As both world demand for animal-sourced foods and global warming concerns rise, safe and effective strategies for enteric methane mitigation in dairy cows are in high demand. This dataset can be used to better understand the efficacy of many potential enteric methane mitigation options.
• This large dataset with descriptive data and treatment means from in vivo dairy cow enteric methane studies can be used by both public and private researchers and advisors including nutritionists, environmentalists, and economists interested in cost-effective solutions to reduce global warming without compromising dairy farm sustainability.
• Evaluation of the data from many enteric methane mitigation experiments with dairy cows can provide indications regarding further research opportunities. Some strategies may prove to have limited research to date. The dataset may also help show potential nutrient and additive interactions needing further consideration and research.

## 1 Objective

Dairy organizations worldwide announced greenhouse gas neutrality goals. Mitigation of enteric methane emissions is necessary to achieve these goals. Many innovative solutions are being tested and considered. A dataset that compiles peer-reviewed, published research results on dietary nutrient reformulation and feed additives and their impact on both enteric methane and dairy cow performance can be used to evaluate promising options for reducing enteric methane emissions from dairy cows while maintaining dairy farm economic sustainability.

## 2 Data Description

One Microsoft Excel file containing manually extracted data from 213 peer-reviewed publications that includes 797 records (rows) and 162 variables (columns). The list of primary data sources is included within the Microsoft Excel file.

## 3 Experimental Design, Materials and Methods

The dataset was created from data collected from scientific publications identified through searches of the scientific literature. The bibliography used by Arndt et al. [1], covering the period from 1963 to 2018, was supplemented with literature searches to cover the period between 2019 and 2022. The Web of Science Core Collection database was searched using the term “methane” in combination with “dairy”, “cattle”, “rumen”, or “ruminant”. Additionally, the search term “rumen” was used in combination with “energy balance”, “energy metabolism”, or “energy partitioning”. A final search was conducted with the terms “enteric” and “dairy”. Publications were included in the corpus when they met the following criteria: English language, detailed production responses, and, at a minimum, enteric methane and dry matter intake means with measures of variance. Data on 797 treatments (rows) from the 213 publications that met the inclusion criteria were manually extracted and compiled into a table containing 162 variables (columns). The variables include publication information, experimental design, animal definition, methane measurement method, and dietary nutrients, as well as treatment means and measures of variance for feed dry matter intake, rumen fermentation parameters, nutrient digestibility, nitrogen excretion, milk and milk components, and enteric methane, carbon dioxide, and hydrogen emissions (Tables 1, 2).

**Table 1:** Description of variables (columns) in the dataset file.

Column	Description	Column	Description	Column	Description
A	Reference number	BL	Rumen Acetate, mol/100 mol	DW	Milk TP, %
B	Publication Title	BM	Rumen Acetate, SEM	DX	Milk TP, SEM
C	Publication Authors	BN	Rumen Acetate, SED	DY	Milk TP, SED
D	Publication Journal	BO	Rumen Acetate, SD	DZ	Milk TP, SD
E	Publication Volume	BP	Rumen Propionate, mol/100 mol	EA	Milk Lactose, %
F	Publication Pages	BQ	Rumen Propionate, SEM	EB	Milk Lactose, SEM
G	Publication Year	BR	Rumen Propionate, SED	EC	Milk Lactose, SED
H	Study Location	BS	Rumen Propionate, SD	ED	Milk Lactose, SD
I	Experimental design	BT	Rumen Butyrate, mol/100 mol	EE	ECM, kg/d
J	Treatment description	BU	Rumen Butyrate, SEM	EF	ECM, SEM
K	Treatment dosage	BV	Rumen Butyrate, SED	EG	ECM, SED
L	Treatment dosage units	BW	Rumen Butyrate, SD	EH	ECM, SD
M	Animals per treatment	BX	Rumen Sampling Method	EI	FCM 3.5%, kg/d
N	Treatment code	BY	TTDM dig, %	EJ	FCM 3.5%, SEM
O	Treatment type	BZ	TTDM dig, SEM	EK	FCM 4%, kg/d
P	Animal metabolic status	CA	TTDM dig, SED	EL	FCM 4%, SEM
Q	Rumen cannulation status	CB	TTDM dig, SD	EM	SCM, kg/d
R	Animal housing type	CC	TTOM dig, %	EN	SCM, SEM
S	Animal feeding type	CD	TTOM dig, SEM	EO	FPCM, kg/d
T	Diet acclimation days	CE	TTOM dig, SED	EP	FPCM, SEM
U	Data collection days	CF	TTOM dig, SD	EQ	FPCM, SED
V	Total treatment days	CG	TTCP dig, %	ER	FPCM, SD
W	Feed analysis method	CH	TTCP dig, SEM	ES	MUN, mg/dL
X	Animal sex	CI	TTCP dig, SED	ET	MUN, SEM
Y	Animal age (months)	CJ	TTCP dig, SD	EU	MUN, SD
Z	Animal BW, kg	CK	TTGE dig, %	EV	CH_4_, g/d
AA	Methane measurement method	CL	TTGE dig, SEM	EW	CH_4_ SEM
AB	DIM	CM	TTGE dig, SED	EX	CH_4_ SED
AC	ADG, kg/d	CN	TTGE dig, SD	EY	CH_4_ SD
AD	ADG, SEM	CO	TTNDF dig, %	EZ	CH_4_/DMI, g/kg
AE	ADG, SD	CP	TTNDF dig, SEM	FA	CH_4_/DMI, SEM
AF	Intake strategy	CQ	TTNDF dig, SED	FB	CH_4_/DMI, SED
AG	Diet forage #1	CR	TTNDF dig, SD	FC	CH_4_/DMI, SD
AH	Diet forage #1, % diet DM	CS	TTADF dig, %	FD	CH_4_, %GEI
AI	Diet forage #2	CT	TTADF dig, SEM	FE	CH_4_, %GEI SEM
AJ	Diet forage #2, % diet DM	CU	TTADF dig, SED	FF	CH_4_, %GEI SED
AK	Supplemental feed ingredients	CV	TTADF dig, SD	FG	CH_4_, %GEI SD
AL	Diet forage, % DM	CW	TTStarch dig, %	FH	CH_4_/Milk, g/kg
AM	DMI, kg/d	CX	TTStarch dig, SEM	FI	CH_4_/Milk, SEM
AN	DMI, SEM	CY	TTStarch dig, SED	FJ	CH_4_/Milk, SED
AO	DMI, SED	CZ	Fecal N, g/d	FK	CH_4_/Milk, SD
AP	DMI, SD	DA	Fecal N, SEM	FL	CH_4_/ECM, g/kg
AQ	Diet GE, Mcal/kg DM	DB	Fecal N, SED	FM	CH_4_/ECM, SEM
AR	Diet CP, %DM	DC	Fecal N, SD	FN	CH_4_/ECM, SED
AS	Diet Fatty Acids, %DM	DD	Urine N, g/d	FO	CH_4_/ECM, SD
AT	Diet EE, %DM	DE	Urine N, SEM	FP	CH_4_/BW Gain, g/kg
AU	Diet Ash, %DM	DF	Urine N, SED	FQ	CH_4_/BW Gain, g/kg, SEM
AV	Diet NDF, %DM	DG	Urine N, SD	FR	H_2_, g/d
AW	Diet ADF, %DM	DH	Milk N, g/d	FS	H_2_, SEM
AX	Diet Lignin, %DM	DI	Milk N, SEM	FT	H_2_, SD
AY	Diet Starch, %DM	DJ	Milk N, SED	FU	H_2_/DMI, g/kg
AZ	Rumen pH	DK	Milk, kg/d	FV	H_2_/DMI, g/kg, SEM
BA	Rumen pH SEM	DL	Milk, SEM	FW	H_2_/DMI, g/kg, SED
BB	Rumen pH SED	DM	Milk, SED	FX	H_2_/DMI, g/kg, SD
BC	Rumen pH SD	DN	Milk, SD	FY	CO_2_, g/d
BD	Rumen Ammonia, mM	DO	Milk Fat, %	FZ	CO_2_ SEM
BE	Rumen Ammonia, SEM	DP	Milk Fat, SEM	GA	CO_2_ SED
BF	Rumen Ammonia, SED	DQ	Milk Fat, SED	GB	CO_2_ SD
BG	Rumen Ammonia, SD	DR	Milk Fat, SD	GC	CO_2_/DMI, g/kg
BH	Rumen VFA, mM	DS	Milk CP, %	GD	CO_2_/DMI, g/kg SEM
BI	Rumen VFA, SEM	DT	Milk CP, SEM	GE	CO_2_/DMI, g/kg SED
BJ	Rumen VFA, SED	DU	Milk CP, SED	GF	CO_2_/DMI, g/kg SD
BK	Rumen VFA, SD	DV	Milk CP, SD

**Table 2:** List of abbreviations used in the dataset file.

Abbreviation	Definition
ADF	Acid Detergent Fiber
ADG	Average Daily Gain (Body Weight)
BW	Body Weight
CGM	Corn Gluten Meal
CH_4_	Enteric Methane
CO_2_	Enteric Carbon Dioxide
CP	Crude Protein (Nitrogen x 6.25)
DDG	Dried Distillers Grains
DDGS	Dried Distillers Grains with Solubles
DIM	Days in Milk (Number of Days Since Calving)
DM	Dry Matter
DMI	Dry Matter Intake
ECM	Energy Corrected Milk
EE	Ether Extract (Fat)
FCM 3.5%	Fat Corrected Milk (3.5%)
FCM 4%	Fat Corrected Milk (4%)
FPCM	Fat and Protein Corrected Milk
GE	Gross Energy
GEI	Gross Energy Intake
H_2_	Enteric Hydrogen
MUN	Milk Urea Nitrogen
N	Nitrogen
NDF	Neutral Detergent Fiber
NIR	Near-Infrared Spectroscopy
OM	Organic Matter
SBM	Soybean Meal
SCM	Solids Corrected Milk
TMR	Total Mixed Ration
TP	True Protein
TTADF dig	Total Tract Acid Detergent Fiber Digested
TTCP dig	Total Tract Crude Protein Digested
TTDM dig	Total Tract Dry Matter Digested
TTGE dig	Total Tract Gross Energy Digested
TTNDF dig	Total Tract Neutral Detergent Fiber Digested
TTOM dig	Total Tract Organic Matter Digested
TTStarch dig	Total Tract Starch Digested
VFA	Volatile Fatty Acids

## Ethics Statements

This study was conducted in compliance with the National Dairy Council ‘Guiding Principles for Research and Communication of Results’ [2].

## CRediT authorship contribution statement

**Mary Beth de Ondarza:** Investigation, Validation, Writing – original draft. **Alexander N. Hristov:** Conceptualization, Project administration, Methodology, Supervision, Validation, Writing – review & editing. **Juan M. Tricarico:** Conceptualization, Funding acquisition, Methodology, Project administration, Supervision, Validation, Writing – review & editing.

## Data Availability

A global dataset of enteric methane mitigation experiments with lactating and non-lactating dairy cows conducted from 1963 to 2022 (Original data) (ScholarSphere (institutional repository managed by Penn State University Libraries)).
